# Methyl 2-{[2,8-bis­(trifluoro­meth­yl)quinolin-4-yl]­oxy}acetate

**DOI:** 10.1107/S1600536811000328

**Published:** 2011-01-12

**Authors:** Zhi-Qiang Feng, Xiao-Li Yang, Yuan-Feng Ye, Huai-Qing Wang, Tao Dong

**Affiliations:** a School of Material Engineering, Jinling Institute of Technology, Nanjing 211169, People’s Republic of China

## Abstract

In the crystal structure of the title compound, C_14_H_9_F_6_NO_3_, mol­ecules are connected by inter­molecular C—H⋯O hydrogen bonds. The best planes through the benzene and pyridyl rings make a dihedral angle of 1.59 (12)°.

## Related literature

The title compound is an important organic synthesis inter­mediate. For the synthetic procedure, see: Lilienkampf *et al.* (2009 ▶). For bond-length data, see: Allen *et al.* (1987 ▶).
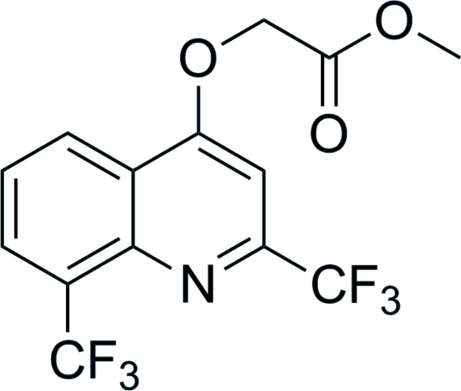


## Experimental

### 

#### Crystal data

C_14_H_9_F_6_NO_3_
*M* *_r_* = 353.22
Monoclinic, 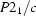

*a* = 4.6980 (9) Å
*b* = 20.549 (4) Å
*c* = 15.176 (3) Å
β = 95.74 (3)°
*V* = 1457.7 (5) Å^3^
*Z* = 4
Mo *K*α radiation
μ = 0.16 mm^−1^
*T* = 293 K
0.30 × 0.20 × 0.10 mm

#### Data collection

Enraf–Nonius CAD-4 diffractometer
Absorption correction: ψ scan (North *et al.*, 1968 ▶) *T*_min_ = 0.953, *T*_max_ = 0.984
3017 measured reflections
2676 independent reflections
1747 reflections with *I* > 2σ(*I*)
*R*_int_ = 0.019
3 standard reflections every 200 reflections  intensity decay: 1%

#### Refinement

*R*[*F* ^2^ > 2σ(*F* ^2^)] = 0.051
*wR*(*F* ^2^) = 0.146
*S* = 1.01
2676 reflections
217 parameters
H-atom parameters constrained
Δρ_max_ = 0.30 e Å^−3^
Δρ_min_ = −0.24 e Å^−3^

### 

Data collection: *CAD-4 Software* (Enraf–Nonius, 1985 ▶); cell refinement: *CAD-4 Software*; data reduction: *XCAD4* (Harms & Wocadlo, 1995 ▶); program(s) used to solve structure: *SHELXS97* (Sheldrick, 2008 ▶); program(s) used to refine structure: *SHELXL97* (Sheldrick, 2008 ▶); molecular graphics: *SHELXTL* (Sheldrick, 2008 ▶); software used to prepare material for publication: *SHELXTL*.

## Supplementary Material

Crystal structure: contains datablocks I, global. DOI: 10.1107/S1600536811000328/vm2070sup1.cif

Structure factors: contains datablocks I. DOI: 10.1107/S1600536811000328/vm2070Isup2.hkl

Additional supplementary materials:  crystallographic information; 3D view; checkCIF report

## Figures and Tables

**Table 1:** Hydrogen-bond geometry (Å, °)

*D*—H⋯*A*	*D*—H	H⋯*A*	*D*⋯*A*	*D*—H⋯*A*
C12—H12*B*⋯O2^i^	0.97	2.54	3.448 (4)	156

Symmetry code: (i) 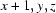.

**Fig. 1.**
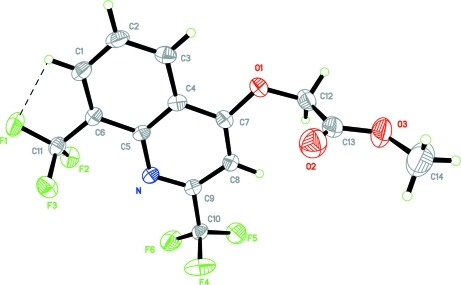
Molecular structure of title compound (I) with atom-numbering scheme. Displacement ellipsoids are drawn at the 40% probability level. Hydrogen bonds are shown by dashed lines.

**Fig. 2.**
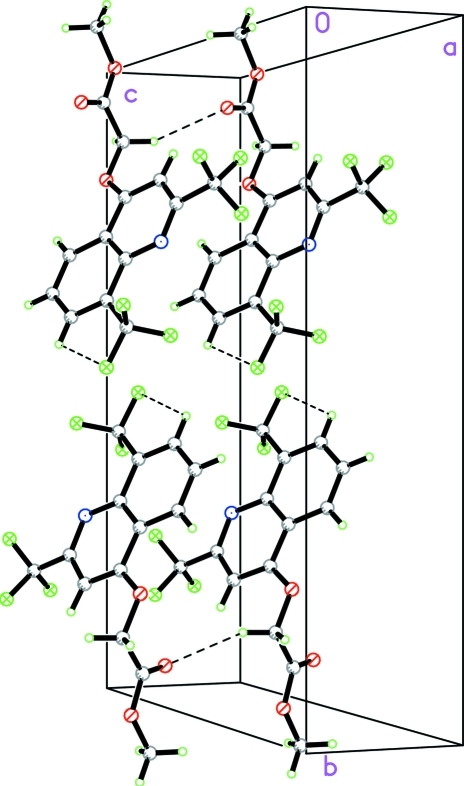
A packing diagram for (I). C—H···O and C—H···F hydrogen bonds are shown by dashed lines.

## supplementary crystallographic information

### Comment

The title compound, methyl 2-((2,8-bis(trifluoromethyl)quinolin-4-yl)oxy)acetate is an important intermediate for the synthesis of drugs (Lilienkampf *et al.*, 2009). Here we report the crystal structure of the title compound, (I).

The molecular structure of (I) is shown in Fig. 1. The bond lengths and angles are within normal ranges (Allen *et al.*, 1987).

The phenyl ring and pyridyl ring are nearly coplanar as indicated by the dihedral angle of 1.59 (12) ° between the best planes through both rings.

The molecules show C—H···O and C—H···F intermolecular and intramolecular hydrogen bonds (Table 1) resulting in a three dimensional network, which seems to be very effective in the stabilization of the crystal structure (Fig. 2).

### Experimental

The title compound, (I) was prepared by a method reported in literature (Lilienkampf *et al.*, 2009). The crystals were obtained by dissolving (I) (0.5 g, 1.42 mmol) in ethanol (25 ml) and evaporating the solvent slowly at room temperature for about 5 d.

### Refinement

All H atoms were positioned geometrically and constrained to ride on their parent atoms, with C—H = 0.93/0.96/0.97 Å for aromatic, methyl and methylene H atoms, respectively, and with *U*_iso_(H) = *xU*_eq_(C), where *x* = 1.2 for aromatic H, and *x* = 1.5 for other H atoms .

### Crystal data

**Table:**

C_14_H_9_F_6_NO_3_	*F*(000) = 712
*M**_r_* = 353.22	*D*_x_ = 1.609 Mg m^−^^3^
Monoclinic, *P*2_1_/*c*	Mo *K*α radiation, λ = 0.71073 Å
Hall symbol: -P 2ybc	Cell parameters from 25 reflections
*a* = 4.6980 (9) Å	θ = 9–13°
*b* = 20.549 (4) Å	µ = 0.16 mm^−^^1^
*c* = 15.176 (3) Å	*T* = 293 K
β = 95.74 (3)°	Block, colourless
*V* = 1457.7 (5) Å^3^	0.30 × 0.20 × 0.10 mm
*Z* = 4	

### Data collection

**Table:**

Enraf–Nonius CAD-4 diffractometer	1747 reflections with *I* > 2σ(*I*)
Radiation source: fine-focus sealed tube	*R*_int_ = 0.019
graphite	θ_max_ = 25.3°, θ_min_ = 1.7°
ω/2θ scans	*h* = 0→5
Absorption correction: ψ scan (North *et al.*, 1968)	*k* = 0→24
*T*_min_ = 0.953, *T*_max_ = 0.984	*l* = −18→18
3017 measured reflections	3 standard reflections every 200 reflections
2676 independent reflections	intensity decay: 1%

### Refinement

**Table:**

Refinement on *F*^2^	Primary atom site location: structure-invariant direct methods
Least-squares matrix: full	Secondary atom site location: difference Fourier map
*R*[*F*^2^ > 2σ(*F*^2^)] = 0.051	Hydrogen site location: inferred from neighbouring sites
*wR*(*F*^2^) = 0.146	H-atom parameters constrained
*S* = 1.01	*w* = 1/[σ^2^(*F*_o_^2^) + (0.080*P*)^2^] where *P* = (*F*_o_^2^ + 2*F*_c_^2^)/3
2676 reflections	(Δ/σ)_max_ < 0.001
217 parameters	Δρ_max_ = 0.30 e Å^−^^3^
0 restraints	Δρ_min_ = −0.24 e Å^−^^3^

### Special details

**Table:**

Geometry. All e.s.d.'s (except the e.s.d. in the dihedral angle between two l.s. planes) are estimated using the full covariance matrix. The cell e.s.d.'s are taken into account individually in the estimation of e.s.d.'s in distances, angles and torsion angles; correlations between e.s.d.'s in cell parameters are only used when they are defined by crystal symmetry. An approximate (isotropic) treatment of cell e.s.d.'s is used for estimating e.s.d.'s involving l.s. planes.
Refinement. Refinement of *F*^2^ against ALL reflections. The weighted *R*-factor *wR* and goodness of fit *S* are based on *F*^2^, conventional *R*-factors *R* are based on *F*, with *F* set to zero for negative *F*^2^. The threshold expression of *F*^2^ > σ(*F*^2^) is used only for calculating *R*-factors(gt) *etc*. and is not relevant to the choice of reflections for refinement. *R*-factors based on *F*^2^ are statistically about twice as large as those based on *F*, and *R*- factors based on ALL data will be even larger.

### Fractional atomic coordinates and isotropic or equivalent isotropic displacement parameters (Å^2^)

**Table:**

	*x*	*y*	*z*	*U*_iso_*/*U*_eq_	
N	0.6586 (4)	0.29913 (10)	0.42412 (14)	0.0383 (5)	
O1	0.5803 (5)	0.19700 (9)	0.65680 (13)	0.0594 (6)	
C1	0.2049 (6)	0.40690 (14)	0.5400 (2)	0.0526 (7)	
H1A	0.1174	0.4474	0.5322	0.063*	
C2	0.1686 (6)	0.37110 (15)	0.61581 (19)	0.0589 (8)	
H2A	0.0538	0.3874	0.6572	0.071*	
O2	0.3296 (6)	0.08697 (12)	0.58771 (19)	0.0863 (8)	
C3	0.2985 (6)	0.31288 (14)	0.62992 (19)	0.0504 (7)	
H3A	0.2751	0.2896	0.6813	0.060*	
O3	0.6519 (6)	0.02604 (12)	0.66729 (19)	0.0956 (9)	
C4	0.4696 (5)	0.28728 (13)	0.56703 (17)	0.0397 (6)	
F4	0.7603 (4)	0.18242 (10)	0.30737 (13)	0.0839 (7)	
F5	1.1459 (4)	0.17660 (9)	0.39252 (12)	0.0840 (7)	
C5	0.5037 (5)	0.32214 (11)	0.48869 (16)	0.0367 (6)	
F6	1.0470 (4)	0.26159 (9)	0.31789 (13)	0.0773 (6)	
C6	0.3659 (5)	0.38388 (11)	0.47696 (17)	0.0398 (6)	
C7	0.6128 (6)	0.22650 (13)	0.57896 (17)	0.0436 (6)	
C8	0.7689 (6)	0.20394 (12)	0.51395 (17)	0.0445 (7)	
H8A	0.8646	0.1643	0.5198	0.053*	
C9	0.7797 (5)	0.24217 (12)	0.43878 (16)	0.0390 (6)	
C10	0.9331 (6)	0.21592 (13)	0.36447 (18)	0.0453 (7)	
C11	0.3985 (6)	0.42315 (13)	0.39586 (19)	0.0462 (7)	
C12	0.7187 (7)	0.13683 (16)	0.6768 (2)	0.0603 (8)	
H12A	0.7505	0.1314	0.7406	0.072*	
H12B	0.9034	0.1365	0.6534	0.072*	
C13	0.5397 (8)	0.08173 (16)	0.6375 (2)	0.0637 (9)	
C14	0.4960 (10)	−0.0321 (2)	0.6392 (3)	0.1169 (16)	
H14A	0.5940	−0.0695	0.6652	0.175*	
H14B	0.4832	−0.0354	0.5759	0.175*	
H14C	0.3070	−0.0301	0.6580	0.175*	
F1	0.2699 (4)	0.48154 (8)	0.39902 (12)	0.0684 (5)	
F2	0.6694 (3)	0.43562 (7)	0.38446 (11)	0.0572 (5)	
F3	0.2832 (3)	0.39444 (8)	0.32177 (11)	0.0619 (5)	

### Atomic displacement parameters (Å^2^)

**Table:**

	*U*^11^	*U*^22^	*U*^33^	*U*^12^	*U*^13^	*U*^23^
N	0.0317 (11)	0.0390 (12)	0.0447 (12)	−0.0009 (9)	0.0068 (9)	−0.0036 (10)
O1	0.0794 (15)	0.0544 (12)	0.0462 (11)	0.0071 (11)	0.0160 (10)	0.0103 (9)
C1	0.0458 (16)	0.0528 (17)	0.0594 (19)	0.0093 (13)	0.0070 (14)	−0.0120 (14)
C2	0.0570 (19)	0.068 (2)	0.0536 (18)	0.0110 (16)	0.0174 (15)	−0.0152 (16)
O2	0.0896 (19)	0.0781 (17)	0.0894 (18)	−0.0053 (15)	−0.0004 (16)	−0.0006 (14)
C3	0.0475 (16)	0.0623 (19)	0.0426 (15)	−0.0016 (14)	0.0108 (13)	−0.0029 (13)
O3	0.120 (2)	0.0559 (15)	0.113 (2)	0.0237 (15)	0.0212 (18)	0.0158 (14)
C4	0.0316 (13)	0.0459 (14)	0.0418 (14)	−0.0052 (11)	0.0050 (11)	−0.0066 (12)
F4	0.0720 (12)	0.1071 (16)	0.0741 (13)	−0.0201 (11)	0.0146 (10)	−0.0478 (11)
F5	0.0813 (14)	0.0987 (15)	0.0746 (13)	0.0501 (12)	0.0215 (11)	0.0041 (11)
C5	0.0287 (12)	0.0400 (13)	0.0419 (14)	−0.0010 (11)	0.0061 (11)	−0.0073 (11)
F6	0.0927 (14)	0.0688 (12)	0.0784 (13)	0.0018 (10)	0.0485 (11)	0.0007 (10)
C6	0.0294 (13)	0.0383 (14)	0.0508 (15)	−0.0013 (11)	−0.0004 (11)	−0.0063 (12)
C7	0.0433 (15)	0.0463 (15)	0.0405 (15)	−0.0025 (12)	0.0014 (12)	0.0038 (12)
C8	0.0471 (15)	0.0401 (14)	0.0461 (16)	0.0037 (12)	0.0040 (12)	−0.0003 (12)
C9	0.0324 (13)	0.0417 (14)	0.0429 (15)	−0.0031 (11)	0.0041 (11)	−0.0037 (12)
C10	0.0442 (15)	0.0437 (15)	0.0483 (16)	0.0059 (13)	0.0072 (13)	−0.0044 (13)
C11	0.0364 (14)	0.0446 (15)	0.0579 (18)	0.0025 (12)	0.0062 (13)	−0.0012 (13)
C12	0.0614 (19)	0.066 (2)	0.0537 (18)	0.0093 (16)	0.0076 (15)	0.0166 (16)
C13	0.078 (2)	0.061 (2)	0.055 (2)	0.0157 (18)	0.0226 (19)	0.0079 (16)
C14	0.138 (4)	0.072 (3)	0.142 (4)	0.001 (3)	0.021 (3)	−0.003 (3)
F1	0.0688 (12)	0.0480 (10)	0.0911 (13)	0.0169 (8)	0.0205 (10)	0.0105 (9)
F2	0.0416 (9)	0.0577 (10)	0.0730 (11)	−0.0068 (7)	0.0090 (8)	0.0047 (8)
F3	0.0648 (11)	0.0651 (11)	0.0537 (10)	−0.0066 (9)	−0.0046 (9)	0.0042 (8)

### Geometric parameters (Å, °)

**Table:**

N—C9	1.311 (3)	F5—C10	1.322 (3)
N—C5	1.362 (3)	C5—C6	1.428 (3)
O1—C7	1.350 (3)	F6—C10	1.320 (3)
O1—C12	1.416 (4)	C6—C11	1.492 (4)
C1—C6	1.362 (4)	C7—C8	1.368 (4)
C1—C2	1.391 (4)	C8—C9	1.390 (3)
C1—H1A	0.9300	C8—H8A	0.9300
C2—C3	1.350 (4)	C9—C10	1.498 (3)
C2—H2A	0.9300	C11—F2	1.327 (3)
O2—C13	1.186 (4)	C11—F3	1.336 (3)
C3—C4	1.410 (4)	C11—F1	1.346 (3)
C3—H3A	0.9300	C12—C13	1.498 (5)
O3—C13	1.321 (4)	C12—H12A	0.9700
O3—C14	1.443 (5)	C12—H12B	0.9700
C4—C5	1.411 (4)	C14—H14A	0.9600
C4—C7	1.421 (4)	C14—H14B	0.9600
F4—C10	1.320 (3)	C14—H14C	0.9600
			
C9—N—C5	116.2 (2)	C8—C9—C10	118.3 (2)
C7—O1—C12	119.4 (2)	F6—C10—F4	106.0 (2)
C6—C1—C2	121.3 (3)	F6—C10—F5	105.8 (2)
C6—C1—H1A	119.3	F4—C10—F5	106.7 (2)
C2—C1—H1A	119.3	F6—C10—C9	113.5 (2)
C3—C2—C1	120.6 (3)	F4—C10—C9	111.8 (2)
C3—C2—H2A	119.7	F5—C10—C9	112.5 (2)
C1—C2—H2A	119.7	F2—C11—F3	106.8 (2)
C2—C3—C4	120.2 (3)	F2—C11—F1	105.8 (2)
C2—C3—H3A	119.9	F3—C11—F1	106.1 (2)
C4—C3—H3A	119.9	F2—C11—C6	113.0 (2)
C13—O3—C14	116.3 (3)	F3—C11—C6	112.9 (2)
C3—C4—C5	120.1 (2)	F1—C11—C6	111.7 (2)
C3—C4—C7	122.4 (2)	O1—C12—C13	110.3 (2)
C5—C4—C7	117.5 (2)	O1—C12—H12A	109.6
N—C5—C4	122.9 (2)	C13—C12—H12A	109.6
N—C5—C6	119.1 (2)	O1—C12—H12B	109.6
C4—C5—C6	117.9 (2)	C13—C12—H12B	109.6
C1—C6—C5	119.9 (3)	H12A—C12—H12B	108.1
C1—C6—C11	120.1 (2)	O2—C13—O3	125.1 (4)
C5—C6—C11	120.0 (2)	O2—C13—C12	125.7 (3)
O1—C7—C8	126.5 (2)	O3—C13—C12	109.2 (3)
O1—C7—C4	114.4 (2)	O3—C14—H14A	109.5
C8—C7—C4	119.1 (2)	O3—C14—H14B	109.5
C7—C8—C9	117.8 (2)	H14A—C14—H14B	109.5
C7—C8—H8A	121.1	O3—C14—H14C	109.5
C9—C8—H8A	121.1	H14A—C14—H14C	109.5
N—C9—C8	126.4 (2)	H14B—C14—H14C	109.5
N—C9—C10	115.3 (2)		
			
C6—C1—C2—C3	1.6 (5)	C4—C7—C8—C9	0.1 (4)
C1—C2—C3—C4	−0.9 (4)	C5—N—C9—C8	−1.1 (4)
C2—C3—C4—C5	−0.6 (4)	C5—N—C9—C10	176.2 (2)
C2—C3—C4—C7	179.5 (3)	C7—C8—C9—N	1.4 (4)
C9—N—C5—C4	−0.7 (3)	C7—C8—C9—C10	−175.9 (2)
C9—N—C5—C6	−180.0 (2)	N—C9—C10—F6	32.0 (3)
C3—C4—C5—N	−177.9 (2)	C8—C9—C10—F6	−150.4 (2)
C7—C4—C5—N	2.1 (3)	N—C9—C10—F4	−87.8 (3)
C3—C4—C5—C6	1.4 (3)	C8—C9—C10—F4	89.8 (3)
C7—C4—C5—C6	−178.7 (2)	N—C9—C10—F5	152.1 (2)
C2—C1—C6—C5	−0.7 (4)	C8—C9—C10—F5	−30.3 (3)
C2—C1—C6—C11	179.4 (2)	C1—C6—C11—F2	123.2 (3)
N—C5—C6—C1	178.5 (2)	C5—C6—C11—F2	−56.7 (3)
C4—C5—C6—C1	−0.8 (3)	C1—C6—C11—F3	−115.5 (3)
N—C5—C6—C11	−1.6 (3)	C5—C6—C11—F3	64.6 (3)
C4—C5—C6—C11	179.1 (2)	C1—C6—C11—F1	4.0 (3)
C12—O1—C7—C8	0.5 (4)	C5—C6—C11—F1	−175.9 (2)
C12—O1—C7—C4	−178.6 (2)	C7—O1—C12—C13	−85.0 (3)
C3—C4—C7—O1	−2.6 (4)	C14—O3—C13—O2	−2.8 (5)
C5—C4—C7—O1	177.5 (2)	C14—O3—C13—C12	176.8 (3)
C3—C4—C7—C8	178.2 (2)	O1—C12—C13—O2	8.6 (4)
C5—C4—C7—C8	−1.7 (4)	O1—C12—C13—O3	−171.1 (3)
O1—C7—C8—C9	−179.0 (2)		

### Hydrogen-bond geometry (Å, °)

**Table:**

*D*—H···*A*	*D*—H	H···*A*	*D*···*A*	*D*—H···*A*
C1—H1A···F1	0.93	2.32	2.675 (3)	102.
C12—H12B···O2^i^	0.97	2.54	3.448 (4)	156

Symmetry codes: (i) *x*+1, *y*, *z*.
